# Transient lesion in the splenium of the corpus callosum due to rotavirus infection

**DOI:** 10.1007/s00381-015-2646-1

**Published:** 2015-02-17

**Authors:** Katarzyna Mazur-Melewska, Katarzyna Jonczyk-Potoczna, Krystyna Szpura, Grzegorz Biegański, Anna Mania, Paweł Kemnitz, Wojciech Służewski, Magdalena Figlerowicz

**Affiliations:** 1 Department of Infectious Diseases and Child Neurology, Karol Marcinkowski University of Medical Sciences in Poznań, Szpitalna Street 27/33, 60-578 Poznań, Poland; 2 Department of Pediatric Radiology, Karol Marcinkowski University of Medical Sciences Poznań, Szpitalna Street 27/33, 60-578 Poznań, Poland

**Keywords:** Rotavirus, Magnetic resonance, Corpus callosum, Child, Transient lesion

## Abstract

Transient signal changes in magnetic resonance imaging (MRI) of the splenium of the corpus callosum (SCC) can result from many different reasons, including encephalitis and encephalopathy caused by infection, seizures, metabolic disorders and asphyxia. We report a case of a 6-year-old Polish girl with rotavirus infection demonstrating a reversible SCC lesion on diffusion-weighted MRI images. She presented six episodes of generalized tonic seizures with mild acute gastroenteritis. Stool test for rotavirus antigen was positive. At the time of admission imaging showed the hyperintense region in T2-weighted and fluid-attenuated inversion-recovery MRI, a well-defined lesion in the splenium of the corpus callosum with restricted diffusion in diffusion-weighted MRI and no enhancement in post contrast T1-weighted imaging. Her first EEG showed slow brain activity in the posterior occipitotemporal portion, consisting mainly of theta waves with a frequency of 4.5–5.5 Hz and amplitude of 40 uV. The lesion had completely disappeared on follow-up MRI 10 days later. The patient recovered fully without any sequelae.

Rotaviruses are responsible for significant gastrointestinal disease, primarily in children <5 years of age. Infection is localized in the intestines but some reports suggest any extraintestinal localization. Recently, a number of investigators have reported central nervous system (CNS) complications in association with rotavirus infections. Neurologic manifestations, ranging from benign convulsions to the viral encephalitis or encephalopathy, occur in approximately 2–5 % of patients with rotavirus gastroenteritis [1–3]. A transient lesion in the splenium of the corpus callosum (SCC) associated with rotaviral infection has been reported in several cases, most of them in patients with encephalopathies. All of them were described in Asian countries like Japan or Korea [4, 5]. There have been no case reports to date of transient SCC lesions associated with rotaviral infections in Europe. We report the first case of a rotavirus gastroenteritis connected with mild encephalitis with a reversible splenial lesion (MERS) in Poland, Europe.

## Case report

A 6-year-old girl was admitted to the hospital after three episodes of generalized, tonic seizures. Two days before admission, she developed watery diarrhoea and repeated vomiting.

Her perinatal history was complicated by premature birth (34th week) with weight 2200 g, Apgar scale −10. Her motor development was delayed in the first 2 years of life.

At admission, the girl complained about moderate abdominal pain and watery stools.

Physical examination showed a body temperature of 37.4 °C, pulse rate of 110 beats/min and blood pressure of 116/60 mmHg. The abdomen was soft, but painful. The bowel sounds were increased. Neurologic examination showed normal condition without pathological reflexes.

Laboratory findings presented the following: leukocyte count 4800/μL, erythrocyte count 4,920,000/μ, platelet count 253,000/μL, haemoglobin 13.3 g/dL and glucose 77 mg/dL. The serum electrolyte levels were as follows: sodium 137 mmol/L, potassium 4.89 mmol/L, calcium 2.49 mmol/L and magnesium 19.3 mmol/L. Aspartate aminotransferase was 49 U/L and alanine aminotransferase 26 U/L. C-reactive protein was elevated to 2.57 mg/dL. Serum samples showed an absence of antibodies against cytomegalovirus, Epstein-Barr, herpes simplex 1 and 2 viruses, *Borrelia burgdorferi* and *Toxoplasma gondii*. Lumbar puncture was not performed; cerebrospinal fluid could not be analysed for this patient.

Enzyme immunoassay detection of Rotavirus in the stool was positive. The stool culture and Adenovirus antigen were negative.

On the day of admission, the girl presented six episodes of generalized tonic seizures with lockjaw and flexion of the extremities. The first two episodes lasted up to 1 min and subsided after diazepam. The subsequent ones were shorter and disappeared spontaneously. Between these episodes, the patient’s consciousness was disturbed. During further hospitalization, no seizures were observed.

**Fig. 1:**
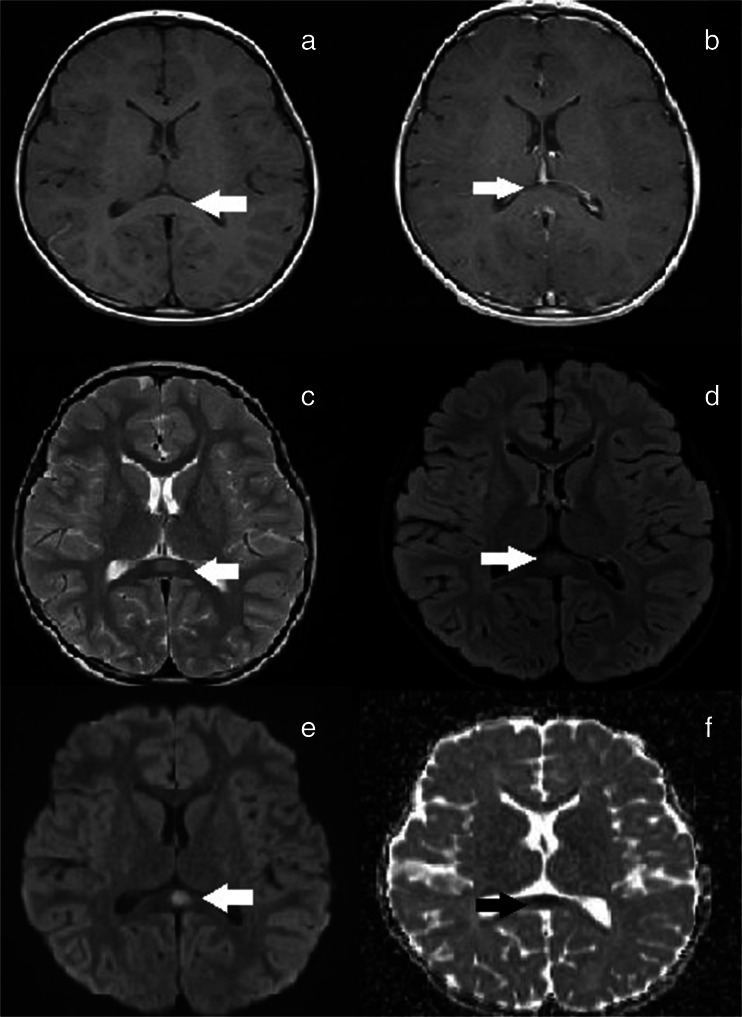
The hyperintense region in T2-weighted and fluid-attenuated inversion-recovery (FLAIR) MRI, a well-defined lesion in the splenium of the corpus callosum with restricted diffusion in diffusion-weighted MRI (DWI) and no enhancement in post contrast T1-weighted (MRI Axial T1W precontrast (**a**) postcontrast (**b**) no lesion enhancement; Axial T2W (**c**), FLAIR (**d**), DWI (**e**) lesion marked using white arrow , ADC (**f**) - black arrow)

Brain magnetic resonance imagination (MRI) was performed on the third day after admission using a 3-T scanner (MAGNETOM Spectra, Siemens Healthcare, Erlangen, Germany). T1-weighted examination was performed before and after i.v. administration of gadobutrol (Bayer Vital, Leverkusen, Germany, 0.1 mmol/kg body weight). The imaging showed the hyperintense region in T2-weighted and fluid-attenuated inversion-recovery (FLAIR) MRI, a well-defined lesion in the splenium of the corpus callosum with restricted diffusion in diffusion-weighted MRI (DWI) and no enhancement in post contrast T1-weighted (Fig. 1).

An electroencephalogram EEG was performed with a 42-channel DigiTrack system (Elmiko, PL), with electrodes placed in compliance with the international 20-electrode system. Her first EEG (the third day) showed slowed brain activity in the posterior occipitotemporal portion, consisting mainly of theta waves with a frequency of 4.5–5.5 Hz and amplitude of 40 uV.

**Fig. 2:**
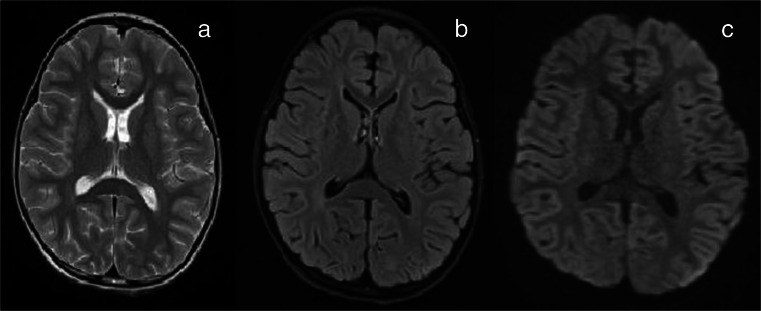
Magnetic resonance imaging (*MRI*) obtained on the tenth day of illness shows complete disappearance of the splenial white matter lesions (MRI Axial T2W (**a**), FLAIR (**b**), DWI (**c**))

She was treated with intravenous crystalloids without anticonvulsant control. After the MRI results, dexamethasone therapy (0.4 mg/kg/day for 5 days) was started.

An MRI performed 12 days after admission revealed no abnormal signal in the splenium of the corpus callosum (Fig. 2). The EEG (awake and sleep) was completely normal.

The child was sent home without signs of neurological complications. At her follow-up examination 2.5 months later, she had age-appropriate motor skills and behaviour. Her EEG showed normal brain activity.

## Discussion

The first observations about the possibility of CNS involvement with rotavirus infection were made by Salmi in 1978. It had been observed in children with rotavirus gastroenteritis with or without associated convulsions, encephalitis and cerebellitis and patients with Reye syndrome and with haemorrhagic brain shock [1, 3, 5]. Our patient manifested recurrent convulsions, confusion and disorientation without electrolyte disturbances. The EEG showed slowed brain activity, in contrast to Isik’s description, who reported reversible sharp waves during rotavirus infection [6]. We confirmed the observation of Kobata et al., who described nine Japanese cases of MERS occurring during rotavirus infection. Among them, 89 % (8/9) had encephalopathic patterns such as alterations in consciousness and abnormal behaviour. Transient SCC lesions have also been reported in encephalitis or encephalopathy induced by other viruses [4]. All reported patients with MERS had a mild clinical course and recovered completely without any sequelae. The mechanism behind these reversible lesions has been analysed by several authors. Tada et al. proposed that the lesions might be secondary to intramyelinic oedema due to disconnection of myelin layers, or else that an incursion of inflammatory cells and macromolecules combined with cytotoxic oedema causes decreased apparent diffusion coefficient levels. This might help to explain the decrease in diffusion levels in the splenium. Splenial lesions after seizures have also been reported in patients with epilepsy. Seizures impair glucose availability, conducted to reversible failure of cellular fluids regulation at the splenium. Reduced diffusion and normalization on follow-up imaging suggest transient intracellular oedema with diffusion restriction [7]. Axonal oedema caused by hyponatraemia or the development of an inflammatory infiltrate were suggested by Takanashi [8]. Oster et al. reported two bitemporal lobe epilepsy patients showing a transient SCC lesion. They suggested the possible mechanism involved a transient disturbance in energy metabolism and ionic transport resulting in reversible myelin vacuolization and intramyelin oedema due to excessive repetitive activity of the commissural projection from the temporal structure in these patients [9]. An analysis performed by Gröppel on 24 epilepsy cases with a focal lesion in the SCC did not present the relationship between the lesion and possible etiologic factors such as epilepsy or seizure types, localization of interictal spikes and ictal EEG patterns or antiepileptic drugs [10]. A reversible SCC lesion could result from multiple conditions including rotaviral infection, gastroenteritis with electrolyte imbalance, seizures, encephalitis and encephalopathy. Taking this into consideration, our patient had many possible factors for the brain lesion’s appearance.

## Abbreviations

ADC: Apparent diffusion coefficient
CNS: Central nervous system
DWI: Diffusion-weighted MRI
EEG: Electroencephalogram
FLAIR: Fluid-attenuated inversion-recovery
MERS: Mild encephalitis with a reversible splenial lesion
MRI: Magnetic resonance imaging
SCC: Splenium of the corpus callosum
